# Human Heart Rhythms Synchronize While Co-sleeping

**DOI:** 10.3389/fphys.2019.00190

**Published:** 2019-03-11

**Authors:** Heenam Yoon, Sang Ho Choi, Sang Kyong Kim, Hyun Bin Kwon, Seong Min Oh, Jae-Won Choi, Yu Jin Lee, Do-Un Jeong, Kwang Suk Park

**Affiliations:** ^1^Interdisciplinary Program in Bioengineering, Seoul National University, Seoul, South Korea; ^2^Department of Neuropsychiatry and Center for Sleep and Chronobiology, Seoul National University Hospital, Seoul, South Korea; ^3^Department of Neuropsychiatry, Eulji University School of Medicine, Eulji General Hospital, Seoul, South Korea; ^4^Department of Biomedical Engineering, College of Medicine, Seoul National University, Seoul, South Korea

**Keywords:** co-sleeping, heart rhythm, phase synchronization, causal relation, non-linear dynamics

## Abstract

Human physiological systems have a major role in maintenance of internal stability. Previous studies have found that these systems are regulated by various types of interactions associated with physiological homeostasis. However, whether there is any interaction between these systems in different individuals is not well-understood. The aim of this research was to determine whether or not there is any interaction between the physiological systems of independent individuals in an environment where they are connected with one another. We investigated the heart rhythms of co-sleeping individuals and found evidence that in co-sleepers, not only do independent heart rhythms appear in the same relative phase for prolonged periods, but also that their occurrence has a bidirectional causal relationship. Under controlled experimental conditions, this finding may be attributed to weak cardiac vibration delivered from one individual to the other via a mechanical bed connection. Our experimental approach could help in understanding how sharing behaviors or social relationships between individuals are associated with interactions of physiological systems.

## Introduction

The structure and function of the cardiac system have traditionally been studied as an independent entity. Recent advances in analytics of non-linear dynamics (Rosenblum et al., 1996; Pikovsky et al., 2003) have identified that the cardiac system interacts with physiological systems under neural regulation (Brandenberger et al., 2001; Rosenblum et al., 2002; Jurysta et al., 2003; Bashan et al., 2012). Synchronization is a phenomenon of adjustment of rhythms due to interaction between periodic or weakly chaotic systems (Pikovsky et al., 2003). The heart rhythm is one of the representative quasiperiodic rhythms generated by the intrinsic cardiac system. Many studies have identified phase synchronization, i.e., emergence of certain relations between the phases and frequencies of interacting systems (Pikovsky et al., 2003), between the cardiac and respiratory rhythms (Schäfer et al., 1998; Bartsch et al., 2007, 2012; Kabir et al., 2010) and between the cardiac and locomotor rhythms (Nomura et al., 2001; Novak et al., 2007). Analysis of the interacting characteristics has led to an understanding of how the cardiac system cooperates with other physiological systems and how this interaction could contribute to physiological homeostasis (Jerath et al., 2014). The concept of synchronization implies that the physiological system of one individual can interact with that of another individual or with an external rhythmic system. One study reported the possibility of phase synchronization between maternal and fetal heart rhythms (Van Leeuwen et al., 2003) and another revealed that the phase synchronization occurs under maternal paced breathing (Van Leeuwen et al., 2009). It has been suggested that the synchronization between maternal and fetal heart rhythms may involve vessel pulsation determined by the maternal heartbeat that is perceived by the auditory system of the fetus (Ivanov et al., 2009; Van Leeuwen et al., 2009). Furthermore, a study that assessed phase synchronization between an internal physiological system and external forces showed that the intrinsic rhythm of the heart can be entrained with periodic visual and auditory stimuli (Anishchenko et al., 2000), but the strength of the stimuli must be strong enough to be recognized. These approaches can provide information on how the physiological system interacts with environmental conditions and changes. The available evidence on adjustment of intrinsic rhythms suggests that interactions between physiological systems could result from neural, mechanical, or behavioral connections.

Sleep is a natural process that plays a major role in restoration (Tononi and Cirelli, 2006; Jerath et al., 2014). There has been much research on the mechanisms of sleep. However, most of the studies have focused on these mechanisms at the level of the individual. In real life, many people sleep beside a partner. Sleep is directly associated with daytime functioning (Bonnet, 1985; Banks and Dinges, 2007) and the risk of chronic diseases (Wolk et al., 2005; Gottlieb et al., 2006; Cappuccio et al., 2008; Tasali et al., 2008). Moreover, there is a possibility that a person's sleep may be influenced by a partner's sleeping behavior (Monroe, 1969; Pankhurst and Home, 1994; Beninati et al., 1999; Edinger et al., 2001; McArdle et al., 2001; Blumen et al., 2012; Drews et al., 2017). Therefore, it is important to be able to understand human sleep in the context of a dyad. A recent study demonstrated significantly more synchronization of sleep stages when couples slept together than when they slept in separate rooms (Drews et al., 2017). Although several studies have investigated co-sleeping patterns (Larson et al., 1991; Meadows et al., 2009; Hasler and Troxel, 2010; Gunn et al., 2015; Richter et al., 2016), there is still limited information available on the level of interaction between the physiological systems of different individuals. During co-sleeping, a mechanical connection is made between individuals via a bed. Ballistocardiography is a measure of the recoil forces of the body caused by ejection of blood from the heart (Starr et al., 1939). This method uses a non-invasive sensor that records the propagation of mechanical vibration through the bed and is widely used for cardiovascular monitoring.

In this study, we investigated the possibility of an interaction between the heart rhythms of co-sleeping individuals. We hypothesized that the degree of phase synchronization of heart rhythms would be greater in co-sleeping individuals than in those who sleep separately and would be attributable to the mechanical connection made between these individuals via the bed. The aim of this research was to determine whether or not there is any interaction between the physiological systems of independent individuals in an environment where they are closely connected with one another.

## Materials and Methods

### Subjects

We analyzed the heart rhythm data for 16 healthy subjects (8 paired individuals) of mean age 27.0 ± 4.3 (range 20–35) years. The subjects consisted of 4 heterosexual married couples and cohabitants and 4 pairs of same-sex friends and co-workers to identify the universality of the interaction between independent physiological systems (Table 1). None of the study participants reported symptoms of a cardiovascular, physical, or mental disorder. The study exclusion criteria were an irregular time schedule within at least 1 week before the experiments, including shift work or international travel that could influence autonomic nervous activity and circadian rhythm, and a self-rated sleep score ≤1. The study protocol was approved by the Institutional Review Board of Seoul National University Hospital and conducted in accordance with the relevant guidelines and regulations. All subjects provided written informed consent before participation in the study.

**Table 1:** Demographic characteristics of the study population.

**Group**	**Relationship**	**Duration (y)**	**Sex**		**Age (y)**		**BMI (kg/m**^****2****^**)**	
1	Co-workers	1.5	M	M	26	28	21.0	22.6
2	Friends	5	F	F	23	24	22.4	18.7
3	Friends	1	F	F	25	23	19.7	21.3
4	Friends	2.5	F	F	20	20	23.2	19.6
Sub-Avg	N/A	2.5	N/A	N/A	23.5	23.8	21.6	20.6
5	Cohabitants	2.5	M	F	30	29	25.7	19.3
6	Married	1	M	F	31	28	23.9	22.1
7	Married	4	M	F	35	33	22.4	20.0
8	Married	1	M	F	29	28	26.0	17.9
Sub-Avg	N/A	2.1	N/A	N/A	31.3	29.5	24.5	19.8
Total-Avg	N/A	2.3	N/A	N/A	27.4	23.6	23.0	20.2

*M, male; F, female; y, years; BMI, body mass index; Avg, average; N/A, not available*.

### Procedure and Measurements

Each pair of individuals performed the experiments at the Center for Sleep and Chronobiology of Seoul National University Hospital. The experiment consisted of two trials, i.e., sleeping together in the same bed and sleeping separately in different beds in the same room. The order of the experiments was randomized. The environmental conditions were controlled for humidity (30–40%) and temperature (24–26°C). The bed in which the paired individuals slept together had a spring-type mattress with dimensions of 200 cm (length) × 150 cm (width) × 25 cm (height). Each pair of individuals visited at ~1 pm on two occasions separated by an interval of 1 week. The experimental protocol and methodology used for data acquisition were explained to all study subjects. An electrocardiogram (ECG) was recorded at the lead II position for each pair of individuals using a wireless device (BN-RSPEC; Biopac Systems, Inc., Goleta, CA, USA). ECG data from paired individuals were synchronously measured using a single analog-to-digital converting system (MP150; Biopac Systems) at a sampling rate of 250 Hz. The subjects were then asked to remain awake for ~20 min of adaptation time, and were allowed to talk to each other during this time. In each trial, the paired individuals slept for 2 h; the average amount of recording time was 122.3 (range, 116.8–128.1) min when the subjects were co-sleeping and 122.9 (range, 111.5–132.5) min when they were sleeping separately (Table 2). We did not perform standard polysomnography; therefore, each subject scored his/her subjective sleep satisfaction from 0 to 5 (0, no sleep at all; 5, very good sleep). The average score reported was 4.1 (range, 2.5–5) during co-sleeping and 3.3 (range, 1.5–4.5) during separate sleeping (Table 2). The ECG was processed with a zero-phase band-pass filter between 0.5 Hz and 35 Hz. R-peak locations were found using an automatic algorithm (Choi et al., 2009) and corrected manually. The heartbeat interval was recorded as the duration between two successive R-peak locations.

**Table 2:** Total recording time and subjective sleep score for experimental trials.

**Group**	**Total recording time (min)**		**Subjective sleep score (0–5)**			
	**Sleeping separately**	**Co-sleeping**	**Sleeping separately**		**Co-sleeping**	
1	123.4	116.8	4.0	3.0	5.0	4.0
2	132.5	128.1	4.0	4.0	4.0	5.0
3	111.5	118.6	3.0	2.0	4.0	4.0
4	125.3	120.9	4.0	3.0	5.0	5.0
Sub-Avg	123.2	121.1	3.8	3.0	4.5	4.5
5	128.4	127.6	4.0	3.0	3.0	4.0
6	119.0	120.3	2.0	3.0	5.0	4.0
7	115.1	119.4	4.0	4.5	3.0	4.0
8	128.3	126.9	1.5	3	4.5	2.5
Sub-Avg	122.7	123.6	2.9	3.4	3.9	3.6
Total-Avg	122.9	122.3	3.3	3.2	4.2	4.1

*min, minute; Avg, average*.

### Interindividual Heart Rhythm Phase Synchronization

**Figure 1:**
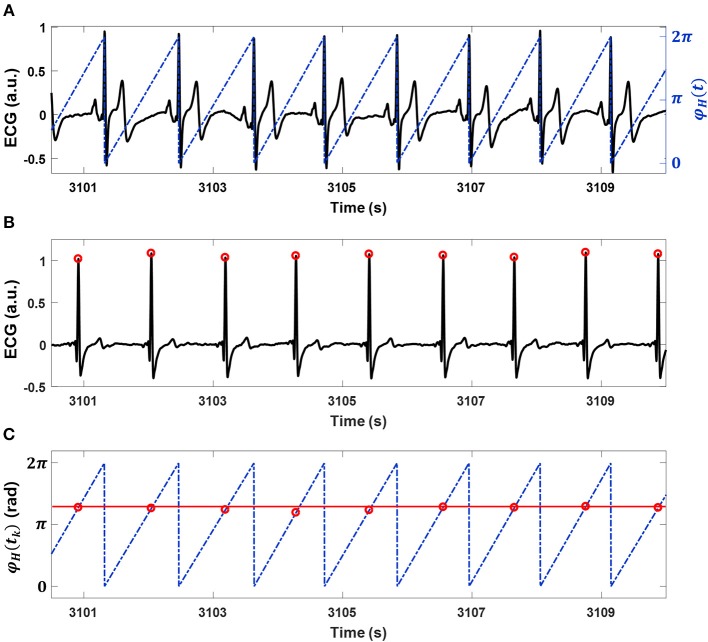
Interindividual heart rhythm phase synchronization (IHPS) and the synchrogram method**. (A)** A subject's electrocardiogram (ECG; black line) with the cyclic phase (blue dashed line) φ_*H*_ (*t*) expressed from 0 to 2π for consecutive R-peak locations. **(B)** An ECG (black line) and its R-peak locations (red dots) for a co-sleeping individual. **(C)** The synchrogram for heart rhythms in a pair of co-sleepers; each R-peak location of the ECG in **(B)** is placed at the corresponding location of the cyclic phase φ_*H*_(*t*) on the ECG in **(A)**. One parallel horizontal line (red line) indicates 1:1 phase synchronization between the heart rhythms. IHPS is determined for different *n*:*m* ratios, where *n* is the number of heartbeats from one individual synchronized with *m* heartbeats from the co-sleeper.

Oscillatory systems adjust their rhythm characteristics when they are linked to interact with each other (Pikovsky et al., 2003). Phase synchronization analysis is used to confirm that the intrinsic frequencies and phases of both systems are locked at a particular ratio of *n*:*m* as a result of their interaction (Bartsch et al., 2012). Therefore, a key feature of interindividual heart rhythm phase synchronization (IHPS) is the consistent occurrence of a series of heartbeats in one individual that are in the same phases of heart rhythm as those in another individual. Phase synchronization can be assessed using a synchrogram (Schäfer et al., 1998) that plots the phase of a first signal (e.g., the phase of one individual's heart rhythm in our study; Figure 1A) at event locations of a second signal (e.g., R-peak locations from other's ECG in our study; Figure 1B) represented by a point process (Bartsch et al., 2012). A synchrogram φ_*H*_(*t*_*k*_) was plotted for the cyclic phase of the heart rhythm in one individual in the pair [φ_*H*_(*t*)] at R-peak locations from the ECG of the other individual in the pair, where *t*_*k*_ denotes the other individual's *k*-th R-peak location (Figure 1C). The cyclic phase was calculated as φ_*H*_(*t*) = Φ _*H*_ (*t*) mod 2π*m*, where Φ _*H*_ (*t*) is the instantaneous phase of heart rhythm defined as follows:

$$\Phi_{H}\left(t\right)=2\pi \cdot \frac{t-t_{i}}{t_{i+1}-t_{i}}+2\pi \cdot i,\;t_{i}\leq t<t_{i+1}$$

where *t*_*i*_ denotes the *i*-th R-peak location.

**Figure 2:**
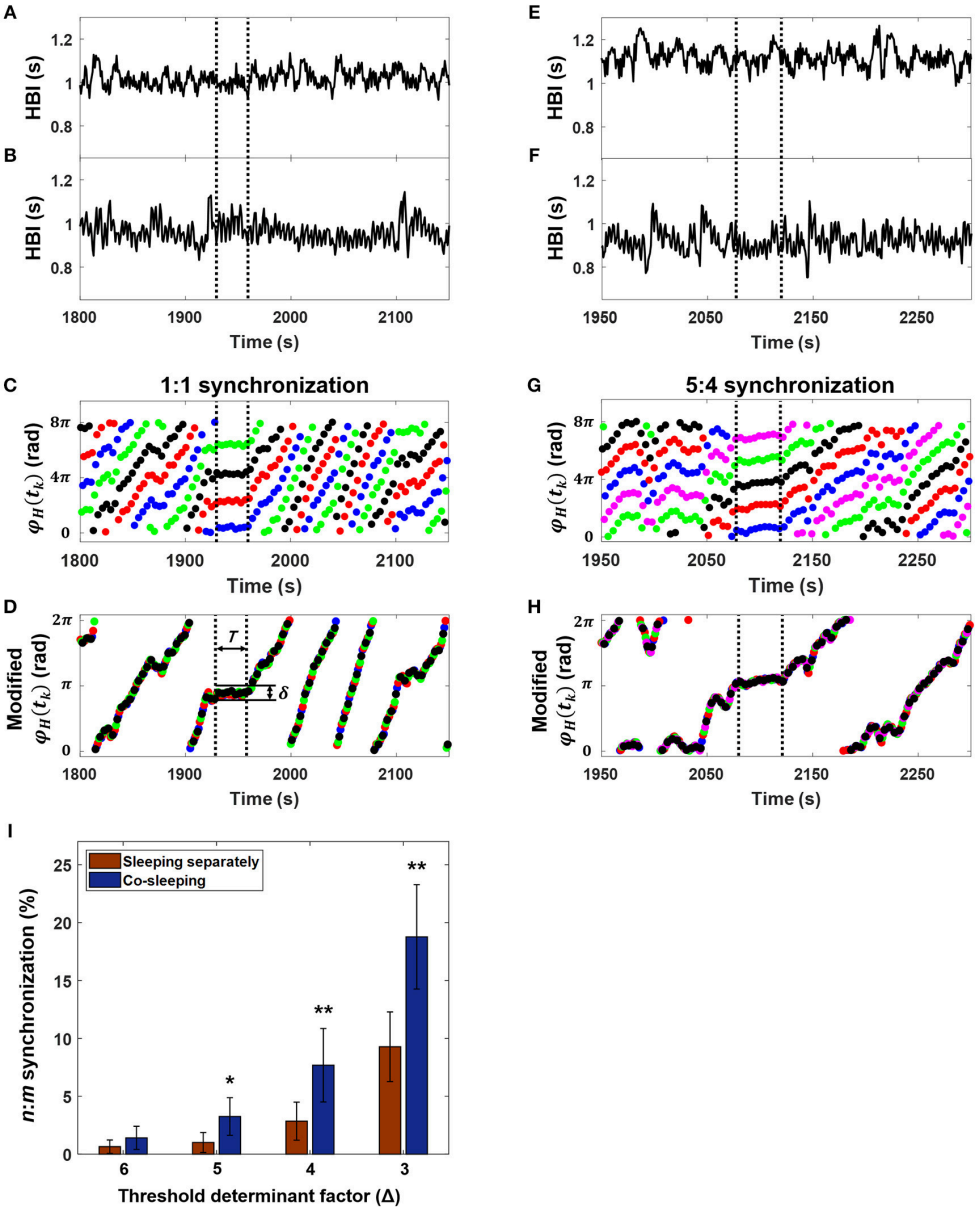
Interindividual heart rhythm phase synchronization (IHPS). **(A)** Heartbeat intervals (HBI) of a subject over 350 s. **(B)** The HBI of a co-sleeper during the same time interval as in **(A)**. **(C)** The corresponding synchrogram φ_*H*_ (*t*_*k*_) with a 1:1 ratio; each colored dot indicates *n* subgrouped heartbeats for the subject over *m* heartbeat cycles for the co-sleeper. **(D)** The synchrogram φ_*H*_ (*t*_*k*_) divided by *m* is then subtracted by each of 2 πρ / *n* where ρ = 1, 2, ···, *n*, and finally wrapped into 0, 2π interval. IHPS (between the vertical dashed lines) is determined for the segment in which variation between the points is lower than the threshold level (δ = 2 π /*n*Δ) maintaining a sufficient time duration (*T*). **(E)** The HBI of another over 350 s. **(F)** The HBI of a co-sleeping individual within the same time interval as that in **(E)**. **(G)** The corresponding synchrogram. **(H)** The synchrogram φ_*H*_ (*t*_*k*_) is expressed using the procedure explained in **(D)** and the IHPS for a 5:4 ratio (between vertical dashed lines). Note that the segments in **(A,B)** and in **(E,F)** were chosen as examples from the entire recordings of co-sleeping individuals to explain the presence of IHPS with different ratios. **(I)** IHPS in different sleep conditions according to the threshold determinant factor (Δ). The degree of IHPS obtained by averaging the percentage of synchronization for each pair of individuals in different sleep conditions. The *n*:*m* ratios are set *m* to 1 ≤ *m* ≤ 10 and *n* to *m* ≤ *n* ≤ *m* + 2. A significantly higher IHPS is observed in co-sleeping individuals (blue bars) than in individuals sleeping separately (red bars), especially at Δ values of 5, 4, and 3. The error bars indicate the standard error. Comparison of the IHPS between co-sleeping individuals and individuals sleeping separately revealed *p < 0.03 at a Δ of 5 and **p < 0.02 at Δ of 4 and 3 (Wilcoxon signed-rank test), indicating that IHPS is significant in co-sleeping individuals when compared with individuals sleeping separately.

Many studies have used automated methods to detect phase synchronization (Kuhnhold et al., 2017). We adapted the method developed by Toledo et al. (2002), which is based on synchrogram analysis. This method has been applied in studies of phase synchronization between fetal-maternal heart rhythms (Van Leeuwen et al., 2003, 2009, 2014). The synchrogram φ_*H*_(*t*_*k*_) determined with the heart rhythms of paired individuals (Figures 2A,B,E,F) was divided into n subgroups alternately (see the color dots in Figures 2C,**G**). For example, the first subgroup includes φ_*H*_(*t*_1_), φ_*H*_(*t*_*n*+1_), φ_*H*_(*t*_2*n*+1_), ···, the second subgroup includes φ_*H*_(*t*_2_), φ_*H*_(*t*_*n*+2_), φ_*H*_(*t*_2*n*+2_), ···, and the *k*-th subgroup contains φ_*H*_(*t*_*n*_), φ_*H*_(*t*_*n*+*k*_), φ_*H*_(*t*_2*n*+*k*_), ···. In phase synchronization, φ_*H*_(*t*_*k*_) represents *n* parallel horizontal lines (Figures 2C,G). For the next step, φ_*H*_(*t*_*k*_) was divided by *m*, after which each subgroup of φ_*H*_(*t*_*k*_) was subtracted by each of 2πρ / *n*, where ρ = 1, 2, ···, *n*, and finally wrapped modulo 2π to eliminate vertical distances between each subgroup of φ_*H*_(*t*_*k*_) (Figures 2D,H). Phase synchronization epochs were found where the variation was maintained lower than τ = 2π /(*n*Δ*)* within *T* = [*t*_*k*_*-*τ/2, *t*_*k*_ + τ/2]. Here, τ was set to 30 s, which was the corresponding window size of sleep analysis, and the Δ value varied from 3 to 6. In the previously reported synchronization analysis of fetal and maternal heart rhythms, the maximum *n* and *m* values were limited to 9 and 4, respectively, because of the difference in basal heart rhythms between the fetus and adult, and the duration of synchronization was set to 10 s (Van Leeuwen et al., 2003). However, the range of heart rhythm in adults is not very different, although the major frequency of heart rhythm could differ because its generation is determined by the intrinsic cardiac system. Therefore, we set *m* to 1 ≤ *m* ≤ 10 and *n* to *m* ≤ *n* ≤ *m*+2; instead, the minimum duration to detect synchronization was 30 s, indicating that ~3 cycles of the *n*:*m* ratio were observed in the case of the maximum ratio (e.g., 11:10), as in the previous fetal-maternal studies. The fluctuation of each heart rhythm could cross over during 2 h of recording. Therefore, the synchronization epochs could not be found in the case of *n* < *m*. To overcome this problem, we also identified the synchronization epochs based on the synchrogram, which was plotted with the use of opposite data (i.e., the cyclic phase in Figure 1B and the R-peaks in Figure 1A). Finally, the degree of IHPS was converted to a percentage of the total duration of synchronization during the time recorded.

### Interindividual Heart Rhythm Causal Relation

**Figure 3:**
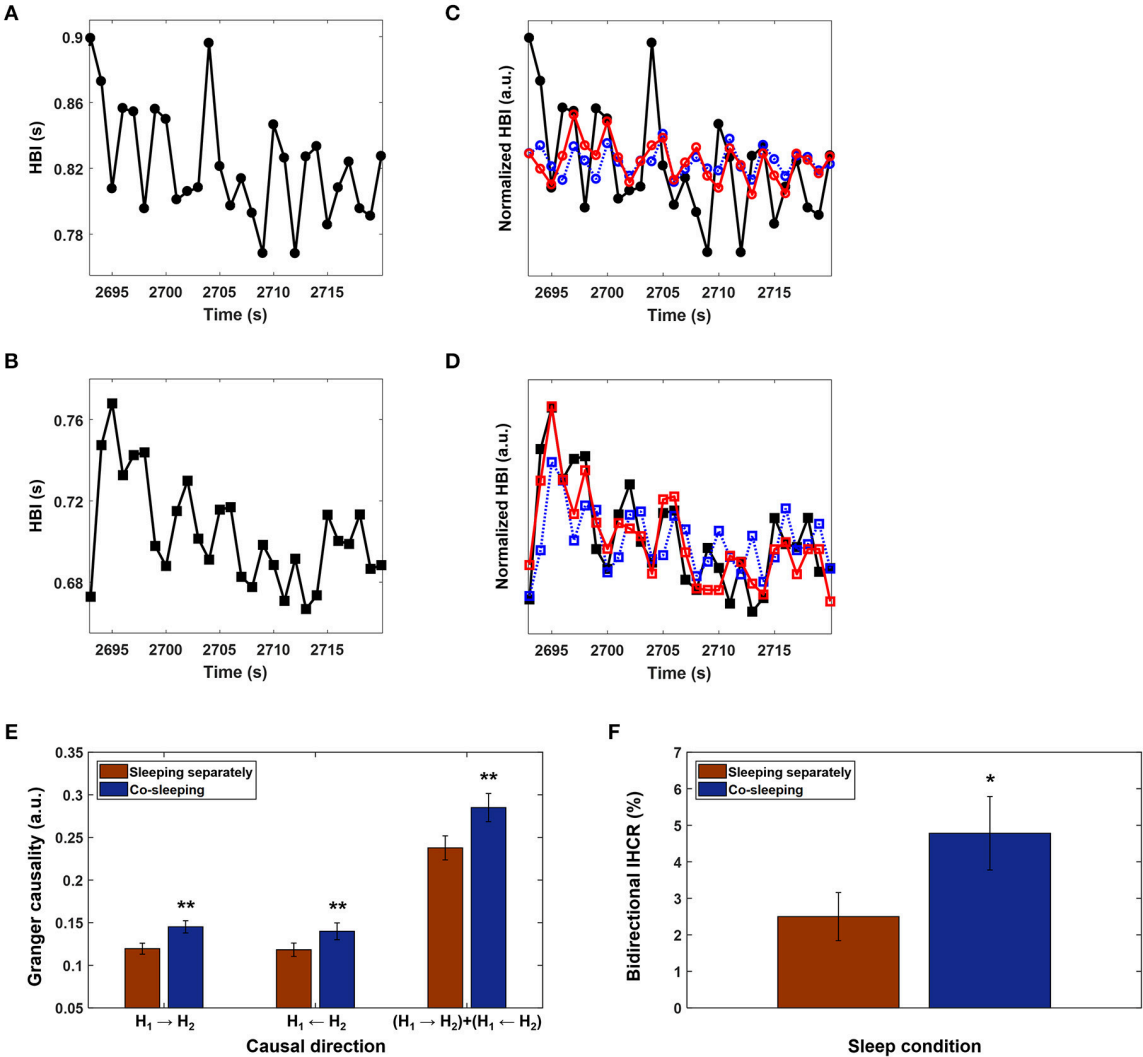
Interindividual heart rhythm causal relation (IHCR). **(A)** and **(B)** Heartbeat intervals (HBI) for a pair of co-sleeping individuals over 30 s that were resampled at 1 Hz to have a corresponding time resolution of 1 s. HBI were chosen as examples to explain the IHCR from the entire recordings of co-sleeping individuals. **(C)** The HBI in **(A)** predicted only using the past HBI in **(A)** (blue dashed line); the corresponding values predicted using both past HBI in **(A)** and **(B)** (red line), but the prediction did not improve. Therefore, the HBI in **(B)** does not have a causal influence on HBI in **(A)**. **(D)** The corresponding procedure described in **(C)** is applied to HBI in **(B)**. In this case, the prediction improves; therefore, the HBI in **(A)** has a causal influence on HBI in **(B)**. **(E)** The mean IHCR obtained by averaging Granger causality for each directional influence of paired individuals' heart rhythms during the entire recordings in different sleep conditions. The error bars indicate the standard error. A significantly higher IHCR in both directions is shown in co-sleeping individuals (blue bars) than in individuals sleeping separately (red bars). Statistical significance is also observed when both directional influences are integrated (**p < 0.02 in all three cases, Wilcoxon signed-rank test). **(F)** The bidirectional IHCR is defined as the percentage of IHCR that is over the threshold (= 0.28) in both directions; this threshold was determined to be twice the average IHCR. The error bars show the standard error. The mean bidirectional IHCR is significantly higher in co-sleeping individuals than in those sleeping separately (*p < 0.03, Wilcoxon signed-rank test), demonstrating that one individual's present heart rhythm is influenced by the other individual's past heart rhythm in both directions during co-sleeping, i.e., the heart rhythms interact with each other.

The causal relation is a different aspect of the interaction between oscillatory systems. The basic aim of causality analysis is to demonstrate whether or not changes in properties are attributable to other influences (Chen et al., 2004). Causal interaction is often assessed by the direction and strength of an interaction; therefore, the influence of one system on another can be quantified. Granger causality is a measure that evaluates causal relations between systems and is calculated using two sets of output time series (Granger, 1969). In our study, these two sets of time series were the heartbeat intervals of paired individuals. Granger causality analysis examines predictive errors to estimate the current value for one system using past measurements for that system as well as the past measurements of both systems (Chen et al., 2004). The other system is deemed to have a causal influence on one system if the prediction error decreases when previous measurements from the other system are included (Figure 3). The interindividual heart rhythm causal relation (IHCR) was evaluated by Granger causality analysis. The heartbeat intervals of the paired individuals were resampled at 1 Hz to have a corresponding time resolution of 1 s. Prediction was performed using resampled heartbeat intervals within 30 s and repeated for heartbeat intervals within the same time window that were shifted by 1 s based on the following equations (Figures 3C,D):

$$\begin{array}{l} \;H_{1}\left(t\right)=\sum_{j=1}^{p}{\text{α}}_{j}\cdot H_{1}\left(t-j\right)+{\text{ε}}_{H_{1}}\left(t\right)\\ H_{1}\left(t\right)=\sum_{j=1}^{p}{\text{β}}_{j}\cdot H_{1}\left(t-j\right)+\sum_{j=1}^{p}{\text{γ}}_{j}\cdot H_{2}\left(t-j\right)+{\text{ε}}_{H_{2}|H_{1}}(t) \end{array}$$

where *H*_1_ and *H*_2_ are heartbeat intervals within 30 s, α, β, and γ denote weighting coefficients, and *p* is the delay factor. The weighting coefficients are determined by autoregressive modeling with the least squares method. The delay factor, *p*, is empirically set to 2, indicating that the current prediction is performed using the data for the previous 2 s. ε_*H*_1__ and ε_*H*_2_|*H*_1__ are the prediction errors when using only the past values of *H*_1_ and when using the past values for both *H*_1_ and *H*_2_, respectively. IHCR from *H*_2_ to *H*_1_ is calculated by the natural logarithm of *var*(ε_*H*_1__)/*var*(ε_*H*_2_|*H*_1__), where *var*(.) denotes the variance. IHCR from *H*_1_ to *H*_2_ is obtained using the same procedure. IHCR was measured using an open source code (Seth, 2010) that was modified for our purpose.

### Analysis of Heart Rate Variability

Heart rate variability (HRV) was measured to evaluate the association between interaction of heart rhythms and autonomic nervous system activity. We observed two time domain HRV parameters, i.e., the standard deviation of heartbeat intervals that were affected by changes in sympathovagal balance and the square root of the mean squared differences of heartbeat intervals associated with parasympathetic activity (Task Force of the European Society of Cardiology the North American Society of Pacing Electrophysiology, 1996; Schmitt et al., 2009). The parameters were calculated using heartbeat intervals during each IHPS and non-IHPS segmented by a non-overlapping window of 30 s in co-sleeping individuals. We obtained the average HRV values (± standard error) for each situation in all subjects.

Sleep status may cause significant differences in the degree of IHPS between co-sleeping and separately sleeping individuals, i.e., low IHPS may occur when a person cannot sleep well or awakens frequently, causing movement. Both situations are characterized by fluctuation in heart rhythm or an increased heart rate (Penzel et al., 2003; Yoon et al., 2018). In this context, the mean heart rate and fluctuation in heart rhythm were measured for each individual and compared between the conditions of co-sleeping and sleeping separately. The mean heart rate was calculated using heartbeat intervals within 30 s of a non-overlapping sliding window in each sleep condition. Fluctuation in heart rhythm was defined as the percentage of the total number of sleep epochs in which the standard deviation of heartbeat intervals was higher than the threshold. In this study, the threshold was decided by twice the average standard deviation of heartbeat intervals during each individual's sleep. We compared the mean (± standard error) heart rate and fluctuations in heart rhythm for each individual during the different sleep conditions.

### Statistical Analysis

The statistical significance of the results for the interaction between heart rhythms in co-sleeping individuals was evaluated by comparing with the results for the interaction between heart rhythms in individuals sleeping separately. A surrogate data test was also performed using the original heartbeat intervals of one individual and a surrogate of the heartbeat intervals of the other individual determined with an amplitude-adjusted Fourier transform algorithm (Theiler et al., 1992). This method provides a surrogate time series with the same distribution of amplitude as the original time series by rescaling the original data and randomizing its Fourier phases to be uniformly distributed between 0 and 2π (Theiler et al., 1992). We analyzed the IHPS and IHCR using same procedure as that described above between the original heartbeat intervals of one individual and 30 surrogates of the other individual's heartbeat intervals in each pair of co-sleepers and separately sleeping individuals.

## Results

### Interindividual Heart Rhythm Phase Synchronization

Threshold determination was a key factor in the analysis. Therefore, we quantified the IHPS by altering the threshold determinant factor (Δ). Our analysis (group mean ± standard error) showed that the degree of IHPS was higher if Δ was from 3 to 6 when a pair of individuals slept in the same bed than when the pair slept in different beds (Figure 2I; Table 3). We found a statistically significant result at Δ values of 3 and 4 and at a Δ of 5 (*p* < 0.02 and *p* < 0.03, respectively, Wilcoxon signed-rank test). Even though the degree of IHPS differed between the different thresholds (Δ), the IHPS value was always at least twice as higher in the co-sleeping condition than in the separate sleeping condition. The longest IHPS durations in the co-sleeping condition were 39.8, 70.2, 182.0, and 344.0 s at Δ values of 6, 5, 4, and 3, respectively.

**Table 3:** Results of interindividual heart rhythm phase synchronization (IHPS) according to Δ variation.

**Group**	**IHPS (%)**							
	**Δ = 6**		**Δ = 5**		**Δ = 4**		**Δ = 3**	
	**Spt**	**Co**	**Spt**	**Co**	**Spt**	**Co**	**Spt**	**Co**
1	0.00	0.00	0.00	0.89	0.00	0.92	6.03	5.81
2	0.00	0.39	0.00	2.00	1.87	5.57	4.02	15.57
3	0.52	0.90	0.57	2.49	2.30	3.79	9.77	10.54
4	0.00	0.00	0.00	0.00	0.00	2.30	4.56	15.74
Sub-Avg	0.13 ± 0.13	0.32 ± 0.21	0.14 ± 0.14	1.35 ± 0.56	1.04 ± 0.61	3.15 ± 1.00	6.1 ± 1.30	11.92 ± 2.37
5	0.00	0.00	0.00	1.30	1.26	6.28	7.01	18.43
6	0.00	1.79	0.48	5.63	3.47	17.03	7.54	39.43
7	4.67	8.22	7.02	13.72	13.92	25.59	29.86	37.00
8	0.00	0.00	0.00	0.00	0.00	0.00	5.44	7.63
Sub-Avg	1.17 ± 1.18	2.5 ± 1.95	1.88 ± 1.72	5.16 ± 3.10	4.66 ± 3.17	12.23 ± 5.68	12.46 ± 5.82	25.62 ± 7.61
Total-Avg	0.65 ± 0.58	1.41 ± 1.00	1.01 ± 0.86	**3.25^b^ ± 1.63**	2.85 ± 1.64	**7.69^a^ ± 3.17**	9.28 ± 3.01	**18.77^a^ ± 4.51**

*The data are shown as the group mean and the standard error. Avg, average; Co, co-sleeping; Spt, sleeping separately. Values for co-sleepers that are significantly different from those for individuals sleeping separately at different levels of Δ are shown in bold*.

a *p < 0.02*;

b *p < 0.03 (Wilcoxon signed-rank test)*.

The statistical significance of the results for IHPS was demonstrated by comparing them with the results for IHPS obtained by surrogate data analysis. The original IHPS result for each group of paired individuals was compared with the results for IHPS calculated using 30 sets of surrogate data from the same group of paired individuals (Table 4). In most co-sleeping individuals, the original IHPS results were significantly higher than those of the surrogate IHPS at different levels of Δ (one-sample *t*-test; see Table 4), except for when the original IHPS was 0%.

**Table 4:** Results of IHPS with surrogate data analysis.

**Group**	**IHPS (%)**											
	**Δ = 6**						**Δ = 5**					
	**Sleeping separately**			**Co-sleeping**			**Sleeping separately**			**Co-sleeping**		
	**Original**	**Surrogate**		**Original**	**Surrogate**		**Original**	**Surrogate**		**Original**	**Surrogate**	
	**Value**	**Average**	**95% CI**	**Value**	**Average**	**95% CI**	**Value**	**Average**	**95% CI**	**Value**	**Average**	**95% CI**
1	0.00	**0.05**^c^	0.00–0.11	0.00	0.00	0.00–0.00	0.00	**0.11**^c^	0.02–0.19	**0.89**^a^	0.22	0.10–0.34
2	0.00	0.00	0.00–0.00	**0.39**^a^	0.13	0.04–0.21	0.00	**0.09**^c^	0.00–0.18	**2.00**^a^	0.53	0.37–0.70
3	**0.52**^c^	0.32	0.16–0.47	**0.90**^b^	0.59	0.41–0.77	0.57	0.77	0.56–0.98	**2.49**^a^	1.48	1.21–1.74
4	0.00	0.00	0.00–0.00	0.00	**0.06**^c^	0.01–0.11	0.00	0.00	0.00–0.00	0.00	**0.11**^c^	0.02–0.21
S. Avg.	0.13 ± 0.13	0.09 ± 0.08	-	0.32 ± 0.21	0.20 ± 0.13	-	0.14 ± 0.14	0.24 ± 0.18	-	1.35 ± 0.56	0.59 ± 0.31	-
5	0.00	**0.05**^c^	0.00–0.10	0.00	**0.06**^c^	0.00–0.11	0.00	**0.23**^a^	0.13–0.33	**1.30**^a^	0.37	0.19–0.54
6	0.00	**0.10**^b^	0.03–0.17	**1.79**^a^	1.11	0.91–1.31	0.48	0.49	0.34–0.65	**5.63**^a^	3.08	2.69–3.48
7	**4.67**^a^	2.64	2.21–3.08	**8.22**^a^	2.91	2.45–3.36	**7.02**^a^	5.30	4.65–5.94	**13.72**^a^	6.40	5.76–7.03
8	0.00	0.00	0.00–0.00	0.00	0.00	0.00–0.00	0.00	**0.11**^c^	0.02–0.20	0.00	0.00	0.00–0.00
S. Avg.	1.17 ± 1.17	0.70 ± 0.65	-	2.50 ± 2.00	1.02 ± 0.68	-	1.88 ± 1.72	1.53 ± 1.26	-	5.16 ± 3.10	2.46 ± 1.48	-
T. Avg.	0.65 ± 0.58	0.40 ± 0.32	-	1.41 ± 1.00	0.61 ± 0.36	-	1.01 ± 0.86	0.89 ± 0.64	-	3.25 ± 1.63	1.52 ± 0.79	-
**Group**	**IHPS (%)**											
	**Δ = 4**						**Δ = 3**					
	**Sleeping separately**			**Co-sleeping**			**Sleeping separately**			**Co-sleeping**		
	**Original**	**Surrogate**		**Original**	**Surrogate**		**Original**	**Surrogate**		**Original**	**Surrogate**	
	**Value**	**Average**	**95% CI**	**Value**	**Average**	**95% CI**	**Value**	**Average**	**95% CI**	**Value**	**Average**	**95% CI**
1	0.00	**0.42^a^**	0.25–0.59	**0.92**^c^	0.67	0.43–0.91	**6.03**^a^	4.00	3.52–4.48	5.81	5.93	5.28–6.59
2	**1.87^a^**	0.37	0.22–0.51	**5.57**^a^	2.09	1.66–2.53	**4.02**^a^	2.28	1.86–2.71	**15.57**^a^	8.86	7.92–9.79
3	2.30	2.18	1.83–2.52	3.79	4.00	3.53–4.48	**9.77**^a^	7.63	6.77–8.49	10.54	**12.09^a^**	11.27–12.91
4	0.00	**0.23^a^**	0.10–0.36	**2.30^a^**	1.28	1.00–1.56	**4.56^a^**	3.09	2.74–3.44	**15.74^a^**	7.32	6.75–7.90
S. Avg.	1.04 ± 0.61	0.80 ± 0.46	-	3.15 ± 1.00	2.01 ± 0.72	-	6.10 ± 1.30	4.25 ± 1.18	-	11.92 ± 2.37	8.55 ± 1.32	-
5	1.26	1.57	1.25–1.89	**6.28**^a^	2.00	1.65–2.35	7.01	**8.52**^a^	7.76–9.28	**18.43^a^**	10.13	9.58–10.69
6	**3.47**^a^	2.28	1.82–2.74	**17.03**^a^	8.65	8.03–9.26	**7.54**^c^	6.53	5.77–7.30	**39.43^a^**	23.61	22.47–24.74
7	**13.92^a^**	11.33	10.45–12.21	**25.59^a^**	14.92	13.86–15.98	**29.86^a^**	24.24	23.24–25.25	**37.00^a^**	29.48	28.56–30.41
8	0.00	**0.36**^a^	0.22–0.50	0.00	**0.17^b^**	0.08–0.28	**5.44**^c^	4.86	4.35–5.37	**7.63^a^**	2.98	2.59–3.36
S. Avg.	4.66 ± 3.17	3.89 ± 2.51	-	12.23 ± 5.68	6.44 ± 3.36	-	12.46 ± 5.82	11.18 ± 4.40	-	25.62 ± 7.61	16.55 ± 6.07	-
T. Avg.	2.85 ± 1.64	2.34 ± 1.32	-	7.69 ± 3.17	4.22 ± 1.80	-	9.28 ± 3.01	7.72 ± 2.48	-	18.77 ± 4.51	12.55 ± 3.25	-

*A one-sample t-test was performed in each group between the results for IHPS obtained with the original data and 30 surrogate data sets. S. Avg and T. Avg are shown as the group mean and standard error. Values that were significantly different in the comparison of results between the original data and surrogate data sets are shown in bold*;

a *p < 0.001*;

b *p < 0.01*;

c *p < 0.05. CI, confidence interval; S. Avg, sub-average; T. Avg, total-average*.

### Interindividual Heart Rhythm Causal Relation

IHCR was evaluated based on Granger causality using the heartbeat intervals within 30 s. We found significant influences in both directions between the heart rhythms of individuals co-sleeping in the same bed and the individuals sleeping separately (Figure 3E; Table 5). We obtained *p* < 0.02 (Wilcoxon signed-rank test) for the IHCR in both directions as well as for the summation of both IHCR. Bidirectional IHCR was defined as the time duration in which the Granger causality values were higher than the threshold in both heart rhythm directions during the total sleep time. The threshold was set to 0.28 which was determined as twice the average IHCR. We found that the bidirectional IHCR was approximately twice as high (*p* < 0.03, Wilcoxon signed-rank test) in co-sleeping individuals than in individuals sleeping separately (Figure 3F, Table 6).

**Table 5:** Results of the average interindividual heart rhythm causal relation (IHCR).

**Group**	**IHCR**					
	**Sleeping separately**			**Co-sleeping**		
	***H_1_*→*H_2_***	***H_1_* ←*H_2_***	**(*H_1_*→*H_2_*)+ (*H_1_* ←*H_2_*)**	***H_1_*→*H_2_***	***H_1_* ←*H_2_***	**(*H_1_*→*H_2_*)+ (*H_1_* ←*H_2_*)**
1	0.103 ± 0.001	0.093 ± 0.001	0.196 ± 0.002	0.123 ± 0.001	0.097 ± 0.001	0.220 ± 0.002
2	0.116 ± 0.001	0.113 ± 0.001	0.229 ± 0.002	0.125 ± 0.002	0.120 ± 0.002	0.245 ± 0.002
3	0.148 ± 0.002	0.146 ± 0.002	0.293 ± 0.003	0.166 ± 0.002	0.145 ± 0.002	0.311 ± 0.003
4	0.113 ± 0.001	0.113 ± 0.001	0.226 ± 0.002	0.161 ± 0.002	0.164 ± 0.002	0.325 ± 0.003
Sub-Avg	0.120 ± 0.010	0.116 ± 0.011	0.236 ± 0.020	0.144 ± 0.011	0.132 ± 0.015	0.275 ± 0.025
5	0.108 ± 0.001	0.106 ± 0.001	0.214 ± 0.002	0.118 ± 0.001	0.116 ± 0.001	0.234 ± 0.002
6	0.101 ± 0.001	0.099 ± 0.001	0.200 ± 0.002	0.168 ± 0.002	0.177 ± 0.002	0.345 ± 0.004
7	0.146 ± 0.002	0.156 ± 0.002	0.302 ± 0.003	0.151 ± 0.002	0.165 ± 0.002	0.316 ± 0.003
8	0.121 ± 0.001	0.120 ± 0.001	0.242 ± 0.002	0.149 ± 0.002	0.135 ± 0.002	0.284 ± 0.003
Sub-Avg	0.119 ± 0.010	0.120 ± 0.013	0.240 ± 0.023	0.147 ± 0.010	0.148 ± 0.014	0.295 ± 0.024
Total-Avg	0.120 ± 0.006	0.118 ± 0.008	0.238 ± 0.014	**0.145^a^ ± 0.007**	**0.140^a^ ± 0.010**	**0.285^a^ ± 0.017**

*The IHCR in each group is shown as the mean and standard error. The Sub-Avg and Total-Avg are shown as the group mean and standard error. Avg, average; H_1_, one individual's heart rhythm; H_2_, the other individual's heart rhythm*.

*Values for co-sleepers that are significantly different from those of individuals sleeping separately are shown in bold*;

a *p < 0.02 (Wilcoxon signed-rank test)*.

**Table 6:** Results for bidirectional IHCR.

**Group**	**Bidirectional IHCR (%)**	
	**Sleeping separately**	**Co-sleeping**
1	0.54	0.99
2	2.02	3.23
3	4.64	5.72
4	2.46	8.53
Sub-Avg	2.42 ± 0.85	4.62 ± 1.62
5	1.53	1.53
6	0.72	8.41
7	5.88	5.65
8	2.21	4.19
Sub-Avg	2.59 ± 1.14	4.95 ± 1.44
Total-Avg	2.50 ± 0.66	**4.78^a^ ± 1.00**

*The data are shown as the group mean and standard error. Bidirectional IHCR is the percentage of IHCR higher than 0.28 in both directions over the total recording time; Avg, average. Values for co-sleepers that are significantly different from those of individuals sleeping separately are shown in bold*;

a *p < 0.03 (Wilcoxon signed-rank test)*.

The significance of the IHCR results was evaluated by comparison with the results for IHCR measured using surrogate data analysis. The results for IHCR from each group of paired individuals were compared with the results for IHCR from 30 sets of surrogate data in the same group of paired individuals (Table 7). In all co-sleeping individuals, the IHCR values were higher in both directions than those from the surrogate data sets (independent-samples *t*-test; see Table 7). Furthermore, the result of bidirectional IHCR was also higher than the results obtained by surrogate data analysis in every pair of co-sleeping individuals (one-sample *t*-test; see Table 8).

**Table 7:** Results for IHCR with surrogate data analysis.

**Group**	**IHCR**											
	**Sleeping separately**						**Co-sleeping**					
	***H*_1_ → *H*_2_**		***H*_1_ ← *H*_2_**		***(H*_1_ → *H*_2_*)*+ *(H*_1_ ← *H*_2_*)***		***H*_1_ → *H*_2_**		***H*_1_ ← *H*_2_**		***(H*_1_ → *H*_2_*)*+ *(H*_1_ ← *H*_2_*)***	
	**Original**	**Surrogate**	**Original**	**Surrogate**	**Original**	**Surrogate**	**Original**	**Surrogate**	**Original**	**Surrogate**	**Original**	**Surrogate**
1	0.103 ± 0.001	**0.107^b^ ± 0.000**	0.093 ± 0.001	0.095 ± 0.000	0.196 ± 0.002	**0.201^b^ ± 0.000**	**0.123^a^ ± 0.001**	0.110 ± 0.000	**0.097^a^ ± 0.001**	0.092 ± 0.000	**0.220^a^ ± 0.002**	0.201 ± 0.000
2	0.116 ± 0.001	0.119 ± 0.000	**0.113^b^ ± 0.001**	0.109 ± 0.000	0.229 ± 0.002	0.228 ± 0.000	**0.125^b^ ± 0.002**	0.121 ± 0.000	**0.120^a^ ± 0.002**	0.111 ± 0.000	**0.245^a^ ± 0.002**	0.231 ± 0.000
3	**0.148^a^ ± 0.002**	0.137 ± 0.000	**0.146^a^ ± 0.002**	0.127 ± 0.000	**0.293^a^ ± 0.003**	0.264 ± 0.000	**0.166^a^ ± 0.002**	0.144 ± 0.000	**0.145^a^ ± 0.002**	0.131 ± 0.000	**0.311^a^ ± 0.003**	0.276 ± 0.000
4	0.113 ± 0.001	**0.116^c^ ± 0.000**	0.113 ± 0.001	**0.118^a^ ± 0.000**	0.226 ± 0.002	**0.234^a^ ± 0.000**	**0.161^a^ ± 0.002**	0.150 ± 0.000	**0.164^a^ ± 0.002**	0.150 ± 0.000	**0.325^a^ ± 0.003**	0.299 ± 0.000
Sub-Avg	0.120 ± 0.010	0.120 ± 0.006	0.116 ± 0.011	0.112 ± 0.007	0.236 ± 0.020	0.232 ± 0.013	0.143 ± 0.011	0.131 ± 0.010	0.132 ± 0.015	0.121 ± 0.012	0.275 ± 0.025	0.252 ± 0.022
5	**0.108^c^ ± 0.001**	0.106 ± 0.000	0.106 ± 0.001	0.106 ± 0.000	0.214 ± 0.002	0.212 ± 0.000	**0.118^a^ ± 0.001**	0.109 ± 0.000	**0.116^a^ ± 0.001**	0.106 ± 0.000	**0.234^a^ ± 0.002**	0.215 ± 0.000
6	0.101 ± 0.001	**0.108^a^ ± 0.000**	0.099 ± 0.001	**0.104^a^ ± 0.000**	0.200 ± 0.002	**0.212^a^ ± 0.000**	**0.168^a^ ± 0.002**	0.133 ± 0.000	**0.177^a^ ± 0.002**	0.142 ± 0.000	**0.345^a^ ± 0.004**	0.275 ± 0.000
7	0.146 ± 0.002	0.146 ± 0.000	0.156 ± 0.002	0.152 ± 0.000	0.302 ± 0.003	0.298 ± 0.000	**0.151^a^ ± 0.002**	0.136 ± 0.000	**0.165^a^ ± 0.002**	0.144 ± 0.000	**0.316^a^ ± 0.003**	0.280 ± 0.000
8	**0.121^a^ ± 0.001**	0.115 ± 0.000	**0.120^a^ ± 0.001**	0.105 ± 0.000	**0.242^a^ ± 0.002**	0.220 ± 0.000	**0.149^a^ ± 0.002**	0.133 ± 0.000	**0.135^a^ ± 0.002**	0.114 ± 0.000	**0.284^a^ ± 0.003**	0.247 ± 0.000
Sub-Avg	0.119 ±0.010	0.118 ±0.009	0.120 ±0.013	0.117 ±0.012	0.239 ±0.023	0.235 ±0.021	0.147 ±0.010	0.128 ±0.006	0.148 ±0.014	0.127 ±0.010	0.295 ±0.024	0.254 ±0.015
Total-Avg	0.120 ± 0.006	0.119 ± 0.005	0.118 ± 0.008	0.114 ± 0.006	0.238 ± 0.014	0.233 ± 0.011	0.145 ± 0.007	0.129 ± 0.005	0.140 ± 0.010	0.124 ± 0.007	0.285± 0.017	0.253 ± 0.012

*The data are shown in each group as the mean and standard error. An independent-samples t-test was performed for each group for results of IHCR obtained with the original data and 30 surrogate data sets. Values that were significantly different in the comparison of results between the original data and surrogate data sets are shown in bold*;

a *p < 0.001*;

b *p < 0.01*;

c *p < 0.05. The Sub-Avg and Total-Avg are shown as the group mean and standard error. Avg, average; H_1_, one individual's heart rhythm; H_2_, the other individual's heart rhythm*.

**Table 8:** Results of bidirectional IHCR with surrogate data analysis.

**Group**	**Bidirectional IHCR (%)**					
	**Sleeping separately**			**Co-sleeping**		
	**Original**	**Surrogate**		**Original**	**Surrogate**	
	**Value**	**Average**	**95% CI**	**Value**	**Average**	**95% CI**
1	0.54	**0.73^b^**	0.62–0.84	**0.99**^a^	0.79	0.69–0.89
2	2.02	1.91	1.74–2.08	**3.23^a^**	2.06	1.88–2.25
3	**4.64^a^**	2.32	2.15–2.49	**5.72^a^**	3.35	3.08–3.61
4	**2.46^a^**	1.67	1.48–1.86	**8.53^a^**	5.60	5.30–5.89
Sub-Avg	2.41 ± 0.85	1.66 ± 0.34		4.62 ± 1.62	2.95 ± 1.03	
5	**1.53^a^**	1.08	0.97–1.18	**1.53^a^**	1.13	1.00–1.26
6	0.72	**0.96^a^**	0.84–1.09	**8.41^a^**	3.50	3.26–3.74
7	**5.88^a^**	5.35	5.06–5.63	**5.65^a^**	4.21	3.89–4.54
8	**2.21^a^**	1.47	1.33–1.62	**4.19^a^**	2.63	2.47–2.79
Sub-Avg	2.58 ± 1.14	2.22 ± 1.05		4.95 ± 1.43	2.87 ± 0.66	
Total-Avg	2.50 ± 0.66	1.94 ± 0.52	–	4.78 ± 1.00	2.91 ± 0.57	—

*Bidirectional IHCR, percentage of IHCR higher than 0.28 in both directions over total recording time. A one-sample t-test was performed in each group between the results for bidirectional IHCR obtained with the original data and 30 surrogate data sets. Values that were significantly different in the comparison of results between the original data and surrogate data sets are shown in bold*;

a *p < 0.001*;

b *p < 0.01. Sub-Avg and Total-Avg are shown as the group mean and standard error. Avg, average; CI, confidence interval*.

## Discussion

In this study, our phase synchronization (IHPS) and causal relation (IHCR) analyses demonstrated a possible interaction between the heart rhythms of co-sleeping individuals. Phase synchronization was used to quantify whether the frequencies and phases of the independent heart rhythms were adjusted to be maintained at a *n*:*m* ratio under a weak interaction. We found that the degree of IHPS was at least twice as high in co-sleeping individuals as that in in individuals sleeping separately at specific thresholds. In most cases, the IHPS was also significantly higher than that obtained by surrogate data analysis, except for the degree of IHPS from the original data representing 0% in each pair of co-sleeping individuals.

**Figure 4:**
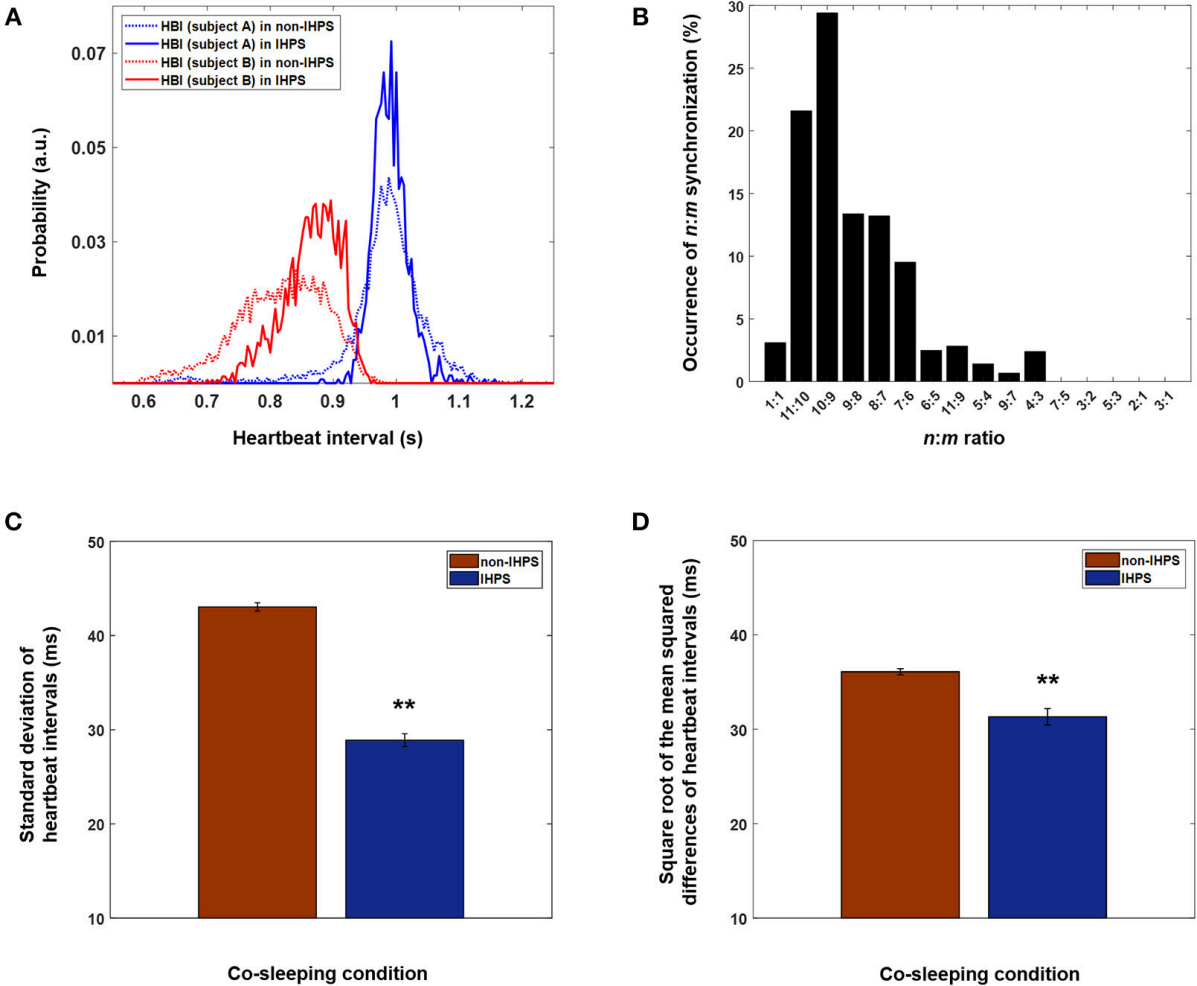
Different *n*:*m* ratios of IHPS at a Δ of 4 and characteristics of heart rhythms in IHPS and non-IHPS in co-sleepers. **(A)** Distributions of heartbeat intervals in IHPS and non-IHPS for a pair of co-sleepers. Narrow and center-concentrated distributions, which are also characterized by a decreased standard deviation of heartbeat intervals, are observed for both heartbeat intervals in IHPS (solid lines) when compared with those in non-IHPS (dashed lines). The distribution of one individual's heartbeat intervals in IHPS (solid red line) shifts in the direction of the co-sleeper's dominant heartbeat interval (solid blue line). In this pair of individuals, the most prevalent *n*:*m* ratio is 10:9. Therefore, one individual's heartbeat intervals are distributed in a particular range where the *n*:*m* ratios are satisfied (around 0.9 s). This indicates that the frequency of the intrinsic rhythm is adjusted because of the interaction, which corresponds to the fundamental characteristics of synchronization. The distributions of heartbeat intervals are determined with a time resolution of 4 ms, which matched the sampling rate (250 Hz) of the recording. The integration of the area in each distribution is 1. **(B)** Various ratios of IHPS close to 1:1 are observed in co-sleeping individuals. The IHPS does not always meet a 1:1 ratio, but different frequency ratios could exist near a 1:1 ratio when their phases are modulated to satisfy the synchronization condition. The histogram is determined with time duration of each *n*:*m* IHPS divided by the total time duration of IHPS observed in all pairs of co-sleeping individuals. **(C)** Standard deviation of heartbeat intervals in IHPS and non-IHPS for co-sleeping individuals. The error bars indicate the standard error. The values are 28.90 ± 0.68 ms and 43.04 ± 0.43 ms in IHPS and non-IHPS, respectively (**p < 0.001, independent-samples *t*-test). **(D)** Square root of the mean squared differences of heartbeat intervals in IHPS and non-IHPS for co-sleeping individuals. The error bars indicate the standard error. The values are 31.32 ± 0.87 ms and 36.09 ± 0.33 ms in IHPS and non-IHPS, respectively (**p < 0.001, independent-samples *t*-test).

The rhythm of oscillatory systems is known to change even when their interaction is weak (Pikovsky et al., 2003). In this study, changes in the distribution of heartbeat intervals were different in IHPS from those in non-IHPS (Figure 4A). In IHPS, the heartbeat interval of one co-sleeper approached the dominant heartbeat interval of the other co-sleeper and was distributed where the *n*:*m* ratio could be established more easily. Moreover, both distributions were more center-concentrated in IHPS than in non-IHPS. The center-concentrated distribution was identified with the standard deviation of heartbeat intervals; this revealed values of 69.3 and 71.6 ms for each individual (blue and red dashed lines, respectively, in Figure 4A) in non-IHPS and of 28.6 and 45.7 ms (blue and red solid lines, respectively, in Figure 4A) in IHPS. These data indicate that the heart rhythms of co-sleeping individuals gradually change in frequency as a result of the interaction and are further synchronized by phase tuning. The difference between heart rhythms in adults is not very large; therefore, a ratio of 1:1 is expected to be the most prevalent ratio of synchronization between heart rhythms in co-sleeping individuals. According to our observations, the synchronization ratio between heart rhythms is close to but not exactly 1:1 (Figure 4B). The heart rhythm is inherently determined by a cardiac system that has complex behaviors with non-linear, non-stationary, and intermittent characteristics (Bashan et al., 2012) causing quasiperiodic oscillations. The frequency of the heart rhythm in one individual can be modulated by interaction with that of another individual, i.e., the heart rhythm of one co-sleeper can act as an external stimulus that affects the heart rhythm of the other co-sleeper. However, the strength of the heart rhythm as a stimulus is not large enough to change the frequency of heart rhythms to meet at exact ratio of 1:1, but it is possible to modulate the frequencies such that they can be maintained at ratios close to 1:1. In these circumstances, phase synchronization of heart rhythms is thought to occur in co-sleeping individuals by fine tuning of their phases at a particular *n*:*m* ratio near the ratio of 1:1. In summary, our experimental results indicate that the heart rhythms of co-sleeping individuals tend to synchronize with ratios closely approaching 1:1 but are not perfectly locked at this ratio. A previous study investigated the synchronization of heart rhythm and periodic visual and auditory stimuli. In that study, the stimulus intervals were determined using own and another individual's average heartbeat intervals in the resting state (Anishchenko et al., 2000). The heart rhythm became synchronized with the external stimuli in both cases, showing 1:1 synchronization when the stimuli interval was generated by own average heartbeat intervals. However, other synchronization ratios, e.g., 7:6, were observed when the stimuli interval was determined from another individual's average heartbeat intervals. Our finding is consistent with the results of a previous study in which intrinsic physiological rhythm became synchronized with another oscillatory rhythm at various ratios close to 1:1. In our study, the heart rhythm of one co-sleeper served as a stimulus for the other co-sleeper, but was relatively small in comparison with the stimuli used in the previous study. The findings of the present research indicate that weak but continuous oscillatory rhythms of independent systems can act as a stimulus for interaction between individuals.

**Figure 5:**
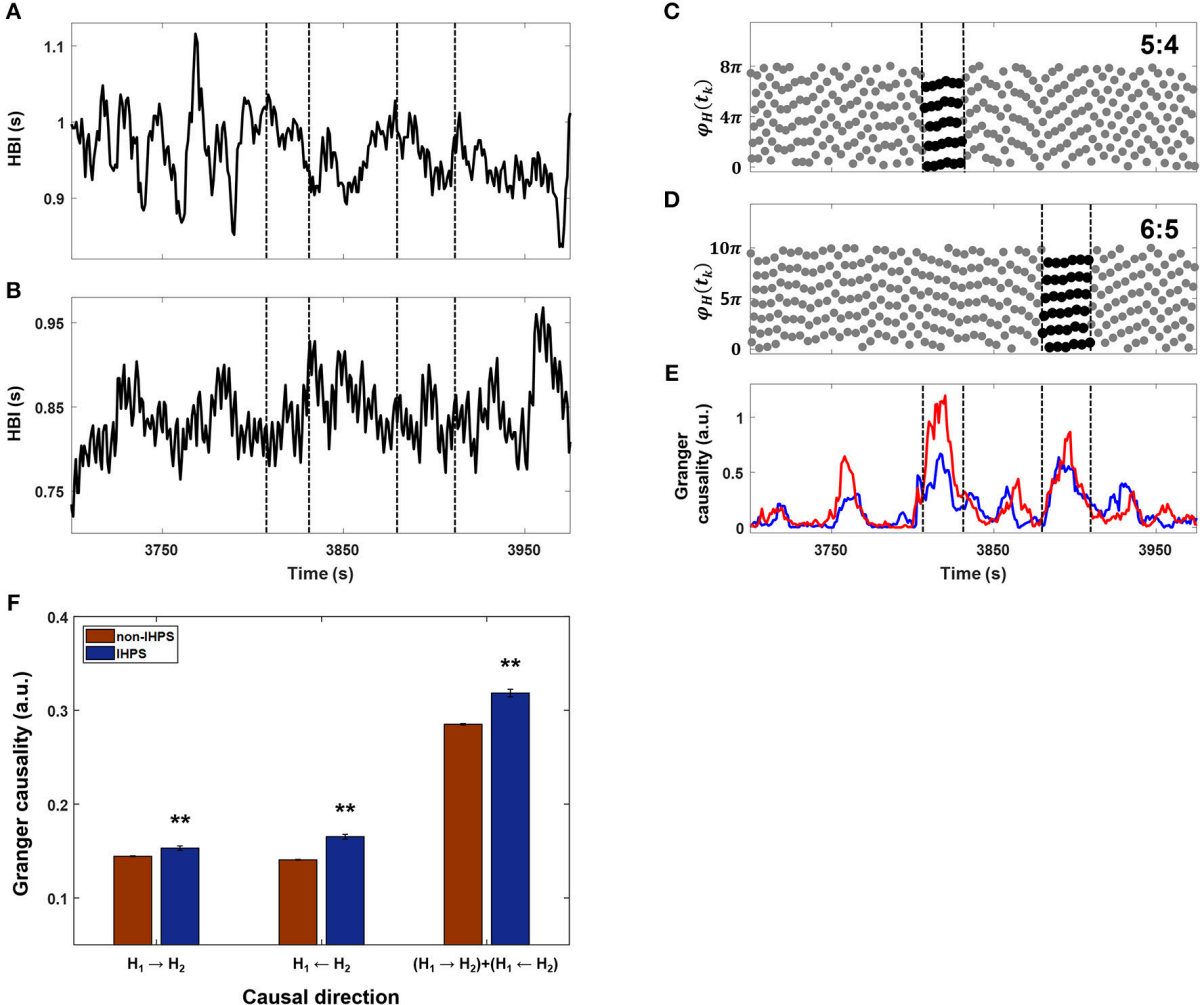
Comparison between IHPS at a Δ of 4 and IHCR. **(A)** The heartbeat intervals (HBI) from one subject for ~250 s. **(B)** The HBI from a co-sleeper for the corresponding time duration. **(C)** The synchrogram determined by heartbeats in **(A)** and **(B)** (gray dots) and IHPS with a ratio of 5:4 (black dots between vertical lines). **(D)** The synchrogram determined by the corresponding heartbeats (gray dots) and IHPS with a ratio of 6:5 (black dots between vertical lines). **(E)** The IHCR directions from HBI in **(A)** to HBI in **(B)** (blue line) and from HBI in **(B)** to HBI in **(A)** (red line). The periods of IHPS with ratios of 5:4 and 6:5 are marked with vertical dashed lines, indicating that IHCR increases in both directions in the IHPS condition. **(F)** Comparison of IHCR between IHPS (blue bars) and non-IHPS (red bars) observed in all co-sleeping individuals during the entire recording. IHCR shows a significant increase in both directions during IHPS. The summation of both directional influences is also significant. The error bars indicate the standard error. The independent samples *t*-test revealed **p < 0.001 for all three cases. IHPS was associated with an increased level of bidirectional influences that provide fine tuning of each frequency and phase.

IHCR analysis revealed that the influence of heart rhythms increased in both directions in co-sleeping individuals. Moreover, bidirectional IHCR was significantly higher in co-sleeping individuals than in individuals sleeping separately. In all cases, the IHCR values were significantly higher in both directions than those from the surrogate data sets in co-sleeping individuals. These findings indicate that the heart rhythms of co-sleepers interact with each other. The IHCR was also evaluated when the IHPS had a Δ of 4 (Figure 5). The results shown in Figure 5F indicate that the IHCR was significantly higher in both directions in IHPS than in non-IHPS (*p* < 0.001, independent-samples *t*-test). Therefore, it appears that the heart rhythms of co-sleepers interact, producing a change in frequency, and that they tend to synchronize by fine tuning of each phase as a result of increasing bidirectional coupling. Briefly, synchronization between heart rhythms is characterized by a bidirectional interaction rather than a one-sided influence of one co-sleeping individual on the other co-sleeping individual.

The analysis in this study was based on heartbeat intervals measured on ECGs recorded in sleeping individuals. HRV provides information about the association with autonomic nervous activity on the basis of sympathetic and parasympathetic tone (Task Force of the European Society of Cardiology the North American Society of Pacing Electrophysiology, 1996). For instance, increased sympathetic tone and parasympathetic withdrawal was reported to lead to increased non-stationary of heartbeat intervals characterized by higher values of standard deviation of heartbeat intervals (Schmitt et al., 2009; Bartsch et al., 2012). We found significantly lower values of the standard deviation of heartbeat intervals in IHPS (28.90 ± 0.68 ms; mean ± standard error) than in non-IHPS (43.04 ± 0.43 ms) for co-sleeping individuals (Figure 4C; *p* < 0.001, independent samples *t*-test). This finding indicates that the synchronization between heart rhythms is associated with autonomic nervous control, particularly sympathovagal balance. The square root of the mean squared differences of heartbeat intervals was smaller in IHPS (31.32 ± 0.87 ms; mean ± standard error) than in non-IHPS (36.09 ± 0.33 ms) for co-sleeping individuals (Figure 4D; *p* < 0.001, independent samples *t*-test). Similar results have been reported for synchronization between maternal and fetal heart rhythms. The square root of the mean squared differences of maternal and fetal heartbeat intervals became smaller with increasing synchronization of maternal and fetal heart rhythms (Van Leeuwen et al., 2009, 2014). Researchers have hypothesized that a more regular maternal heartbeat generates a more stable acoustic stimulus, which allows the fetal heartbeat to become better synchronized (Ivanov et al., 2009; Van Leeuwen et al., 2009).

IHPS is considered to occur during fluctuations in both heart rhythms triggered by movements or sleep-related events in one of the co-sleepers, such as snoring or sleep apnea. However, such events and movements are associated with increased sympathetic nervous activity (Somers et al., 1995; Lanfranchi and Somers, 2011), which is represented by an increased standard deviation of heartbeat intervals (Penzel et al., 2003; Schmitt et al., 2009). We observed that the standard deviation of heartbeat intervals during IHPS in co-sleeping was low. Therefore, IHPS is unlikely to occur in such situations, but could occur when both heart rhythms are stable. These characteristics have been found in phase synchronization between physiological systems, such as the cardiorespiratory system in the same individual during sleep, i.e., the degree of synchronization is lower in the awake state than during light or deep sleep (Bartsch et al., 2007, 2012). Moreover, the degree of cardiorespiratory synchronization is lower in patients with obstructive sleep apnea than in healthy subjects during sleep (Kabir et al., 2010).

Aside from the sleeping conditions (co-sleeping vs. separate sleeping), the degree of IHPS can be influenced by sleep status, such as frequent awakening because of movement and remaining awake, which is characterized with an increased heart rate and fluctuation in heart rhythm. In our study, the degree of fluctuation in heart rhythm was determined by the percentage of segments for which the standard deviation of heartbeat intervals was higher than that of threshold (i.e., twice the average value) during the entire sleep recording. We found that the degree of fluctuation in heart rhythm was not significantly different between co-sleeping individuals (4.09% ± 0.60%; mean ± standard error) and individuals sleeping separately (3.28 ± 0.61%; *p* > 0.05, Wilcoxon signed-rank test; Table 9). Furthermore, the mean heart rate was not significantly different between co-sleeping individuals (67.32 ± 1.90 beats per minute) and individuals who slept separately (67.82 ± 2.43 beats per minute; *p* > 0.05, Wilcoxon signed-rank test; Table 10). Therefore, the sleep status in co-sleeping and separately sleeping individuals is not significantly different, at least in heart rhythm variation which may be associated with the degree of IHPS.

**Table 9:** Results of fluctuation in heart rhythm in different sleep conditions.

**Group**	**Fluctuation in heart rhythm (%)**			
	**Sleeping separately**		**Co-sleeping**	
1	2.44	1.63	2.58	1.29
2	2.64	2.26	5.47	1.56
3	3.59	4.04	9.28	5.49
4	1.20	2.80	2.90	2.07
Sub-Avg	2.47 ± 0.49	2.68 ± 0.51	5.06 ± 1.55	2.60 ± 0.98
5	2.73	2.34	1.18	3.53
6	6.72	3.36	6.67	2.92
7	10.43	4.78	6.72	6.72
8	1.17	0.39	2.77	4.35
Sub-Avg	5.26 ± 2.08	2.68 ± 0.92	4.34 ± 1.40	2.60 ± 0.83
Total-Avg	3.87 ± 1.12	2.70 ± 0.49	4.70 ± 0.98	3.49 ± 0.68
	3.28 ± 0.61		4.09 ± 0.60	

*Sub-Avg and Total-Avg are shown as the group mean and standard error. There was no statistically significant difference in the fluctuation in heart rhythm between co-sleepers and individuals sleeping separately (p > 0.05, Wilcoxon signed-rank test)*.

*Avg, average*.

**Table 10:** Mean heart rate according to sleep conditions.

**Group**	**Mean heart rate (bpm)**			
	**Sleeping separately**		**Co-sleeping**	
1	65.41 ± 0.35	64.32 ± 0.27	65.12 ± 0.29	62.81 ± 0.29
2	63.88 ± 0.35	86.50 ± 0.26	67.13 ± 0.53	72.55 ± 0.23
3	71.76 ± 0.52	71.05 ± 0.37	74.71 ± 0.37	70.59 ± 0.31
4	68.64 ± 0.28	83.22 ± 0.54	74.28 ± 0.26	86.17 ± 0.33
Sub-Avg	67.42 ± 1.75	76.27 ± 5.19	70.31 ± 2.45	73.03 ± 4.86
5	62.77 ± 0.28	60.99 ± 0.38	68.36 ± 0.42	56.21 ± 0.23
6	79.61 ± 0.35	77.64 ± 0.28	60.97 ± 0.19	72.76 ± 0.36
7	55.77 ± 0.27	55.63 ± 0.20	58.52 ± 0.30	62.00 ± 0.22
8	61.41 ± 0.39	56.53 ± 0.26	62.99 ± 0.28	61.90 ± 0.23
Sub-Avg	64.89 ± 5.14	62.70 ± 5.12	62.71 ± 2.09	63.22 ± 3.46
Total-Avg	66.16 ± 2.56	69.49 ± 4.24	66.51 ± 2.07	68.12 ± 3.33
	67.82 ± 2.43		67.32 ± 1.90	

*The data are shown in each group as the mean and standard error. The Sub-Avg and Total-Avg are shown as the group mean and standard error. There was no statistically significant difference in the mean heart rate between co-sleepers and individuals sleeping separately (p > 0.05, Wilcoxon signed-rank test)*.

*Avg, average*.

Our results demonstrate that co-sleeping in the same bed provides environmental conditions for interaction between heart rhythms. We attribute this interaction to ballistocardiographic vibration (Starr et al., 1939) through the bed. In contrast with a previous study (Anishchenko et al., 2000), this level of stimulus is so weak that most people do not recognize its existence. The bed acts as a medium to connect both co-sleepers' cardiac systems and delivers their small mechanical vibrations that allow the systems to interact (Supplementary Video 1). Therefore, characteristics of the bed that are associated with delivering vibrations, such as elasticity and hardness, can influence the occurrence of IHPS in co-sleeping individuals, which were controlled for in this study (all participants slept in the same beds for different sleeping conditions). Our study provides objective information on how independent cardiac systems interact with each other when individuals co-sleep. This phenomenon is under the control of the autonomic nervous system. Further studies are needed to investigate whether or not the interaction influences on sleep architecture which is a well-defined condition of autonomic nervous activity (Penzel et al., 2003; Schmitt et al., 2009; Bartsch et al., 2012). Our observations indicate that synchronization of heart rhythms varies widely between co-sleeping individuals and may be influenced by various factors. Exactly how different physiological conditions and states (Schmitt et al., 2009; Bartsch et al., 2012) are associated with the degree of interaction warrants further investigation. In several groups of individuals who slept separately, the original IHPS and IHCR showed higher values than the results for surrogate IHPS and IHCR, even though the results from the original data were expected to show no significant differences or to be lower than those from the surrogate data set. More studies are needed to identify other factors that may influence the interaction between heart rhythms of sleeping individuals, such as the sleep stage-dependent heart rhythm pattern.

Co-sleeping is a shared behavior that takes up one third of the time a couple spend together. Therefore, various studies have investigated concordance in wake-sleep patterns between couples and its association with attachment style, marital satisfaction, and the functioning of the relationship during the day time (Larson et al., 1991; Meadows et al., 2009; Hasler and Troxel, 2010; Gunn et al., 2015; Richter et al., 2016). Analysis of the interaction between physiological systems could provide more information on the relationship between sleep concordance and marital satisfaction.

Our findings highlight an unconscious manner in which individuals communicate with each other. Individuals might have communicated and interacted with each other by weak coupling that they do not recognize. Whether or not one individual's cardiac rhythm, which is normally under intrinsic control, can be controlled by an another individual's physiological rhythm or an external non-physiological rhythm even under weak coupling needs further study. We believe such research will provide a new paradigm that affects, improves, and controls the autonomic rhythm of others by induced coupling even in unrecognizable intensity.

## Data Availability

The raw data supporting the conclusions of this manuscript will be made available by the corresponding author, without undue reservation, to any qualified researcher upon reasonable request.

## Ethics Statement

The study protocol was approved by the Institutional Review Board of Seoul National University Hospital (No. 1607-094-776) and conducted in accordance with the tenets of the Declaration of Helsinki. All subjects provided written informed consent.

## Author Contributions

HY, SHC, SKK, and KSP designed the research. HY, SHC, and HBK performed the research. HY and SKK contributed analytic tools. HY, SHC, HBK, SMO, J-WC, YJL, D-UJ, and KSP analyzed the data. HY and KSP wrote the paper

### Conflict of Interest Statement

The authors declare that the research was conducted in the absence of any commercial or financial relationships that could be construed as a potential conflict of interest.

## Supplementary Material

Supplementary Video 1 Synchronization of metronomes on a bed.
